# Complex evolution of the *DAL5 *transporter family

**DOI:** 10.1186/1471-2164-9-164

**Published:** 2008-04-11

**Authors:** Linda Hellborg, Megan Woolfit, Mattias Arthursson-Hellborg, Jure Piškur

**Affiliations:** 1 Cell and Organism Biology, Lund University, Lund, Sweden; 2 Smurfit Institute of Genetics, Trinity College, University of Dublin, Ireland; 3 School of Integrative Biology, University of Queensland, Brisbane, Queensland, 4072, Australia; 4 Jayway AB, Malmö, Sweden

## Abstract

**Background:**

Genes continuously duplicate and the duplicated copies remain in the genome or get deleted. The *DAL5 *subfamily of transmembrane transporter genes has eight known members in *S. cerevisiae*. All are putative anion:cation symporters of vitamins (such as allantoate, nicotinate, panthotenate and biotin). The *DAL5 *subfamily is an old and important group since it is represented in both Basidiomycetes ("mushrooms") and Ascomycetes ("yeast"). We studied the complex evolution of this group in species from the kingdom of fungi particularly the Ascomycetes.

**Results:**

We identified numerous gene duplications creating sets of orthologous and paralogous genes. In different lineages the *DAL5 *subfamily members expanded or contracted and in some lineages a specific member could not be found at all. We also observed a close relationship between the gene YIL166C and its homologs in the *Saccharomyces sensu stricto *species and two "wine spoiler" yeasts, *Dekkera bruxellensis *and *Candida guilliermondi*, which could possibly be the result of horizontal gene transfer between these distantly related species. In the analyses we detect several well defined groups without *S. cerevisiae *representation suggesting new gene members in this subfamily with perhaps altered specialization or function.

**Conclusion:**

The transmembrane *DAL5 *subfamily was found to have a very complex evolution in yeast with intra- and interspecific duplications and unusual relationships indicating specialization, specific deletions and maybe even horizontal gene transfer. We believe that this group will be important in future investigations of evolution in fungi and especially the evolution of transmembrane proteins and their specialization.

## Background

Transmembrane transporters of unicellular organisms, like yeast, are one of the primary links between the outer world and the metabolic pathways inside the cell. The importance of these genes is seen in the substantial proportion of transporter genes within the yeast genome (10%) [1]. In *Saccharomyces cerevisiae*, for example, over 400 genes encoding transporter proteins have been found [2].

Different species of yeast require different substrates to be transported, and the number and kind of transporters present in the genome therefore vary between species. The presence or absence of a transporter may provide insight into the niche preference and metabolic ability of a yeast [3-6]. An example is seen in the loss or inactivation of the galactose transporter Gal2p (and six other genes of the galactose metabolism) in several Hemiascomycete species [7]. Similarly, expansion or contraction of gene numbers in various transporter subfamilies can indicate metabolic abilities: for example, peroxisomal and long chain fatty acid transporters have undergone amplification in *Y. lipolytica*, which is known to grow on fatty acids [8]. More unexpectedly, genes belonging to two heavy metal transporter subfamilies, SIT and CT2, have been amplified 14 and 10 times respectively in *Y. lipolytica *[9], possibly as the result of a natural symbiosis with bacteria providing iron-siderophores and/or other natural chelators.

Yeast transporters have been classified into a number of families and subfamilies, based on functional and phylogenetic criteria [8,10]. Within the Hemiascomycete phylum, 97 small phylogenetic transporter subfamilies have been identified comprising a total of 355 transporters named according to their evolutionary patterns ("ubiquitous," "species specific," "phylum gains and losses," and "homoplasic")[8].

The subfamily which is most variable in gene number across yeast species is the anion:cation symporter subfamily (TC number 2.A.1.14) of the Major Facilitator Superfamily [8]. These transporters are all putative weak acid permeases, which take up anionic vitamins (allantoate, nicotinate, panthotenate, biotin and thiamine) in symport with protons or cations. In *Saccharomyces cerevisiae *the allantoate permease family consists of eight proteins where the allantoate permease YJR152w (*DAL5*) has given its name to the subfamily. The other members of this family are YLR004c (*TH173*), YLL055w (*YCT1*), YGR260w (*TNA1*), YIL166c, YAL067c (*SEO1*), YGR065c (*VHT1*), YCR028c (*FEN2*) [10] (Table 1).

**Table 1:** [8]-Gene name and function of the eight members of the *DAL5 *subfamily in *S. cerevisiae*.

Gene name	Function	References
YJR152w (*DAL5*)	Encodes an allantoate and ureidosuccinate permease, expression is constitutive but sensitive to nitrogen catabolite repression. Subtelomeric in *S. cerevisiae*	[20, 21].
YCR028c (*FEN2*)	A membrane pantothenate transporter, regulated by high concentrations of pantothenate. Pantothenate is essential for the biosynthesis of coenzyme A, which is a carrier of activated C2 units in sterol biosynthesis. *FEN2 *was first identified in a screen for mutants resistant to fenproprimorph, an inhibitor of ergosterol biosynthesis	[22, 23]
YGR065c (*VHT1*)	Encodes a high affinity H^+^-biotin (vitamin H) permease different from mammals, regulated by high concentrations of biotin. The biotin uptake and biosynthesis is reciprocally regulated by iron, with uptake being activated when iron is scarce	[22, 24].
YGR260w (*TNA1*)	Encodes a high affinity nicotinic acid (vitamin B3) permease. The mRNA levels increase strongly at reduced extracellular concentrations of both nicotinic acid and para-aminobenzoate (PABA) but are not inhibited by high concentrations of the same substrates. Subtelomeric in *S. cerevisiae*	[25, 26].
YLR004c (*TH173*)	Encodes a membrane protein in the endoplasmic reticulum that is strongly regulated by thiamine. It is unable to transport thiamine but might be involved in transport of thiamine precursors. The metabolite is probably a compound that can be used by yeast to generate the thiazole precursor HET. The expression level is upregulated by Pdc2 (pyruvate decarboxylase) and Thi2 (thiamine)	[10, 26-30].
YAL067c (*SEO1*)	Might be involved in the transport of some sulphur compound (which remains to be identified) since the overexpression of the *SEO1 *gene allowed growth on low concentration of methionine sulphoxide and supressed the ethionine sulphoxide resistance. The gene does not encode a methionine permease Subtelomeric in *S. cerevisiae*	[31].
YLL055w (*YCT1*)	Might be involved in the intracellular transport of allantoate since its transcripts are overexpressed during nitrogen starvation (similar to *DAL5*) The protein is localized in the endoplasmic reticulum. Subtelomeric in *S. cerevisiae*	[32].
YIL166c	Putative protein with elevated mRNA expression by sulfur limitation. YIL166c is a non-essential gene. Subtelomeric in *S. cerevisiae*	[10, 27-29].

To investigate the evolution of this gene family, and track its expansions and contractions across Hemiascomycetes, we have performed a phylogenetic analysis of these genes across a number of Hemiascomycete species for which whole genomes are available. The *DAL5 *subfamily is a ubiquitous transporter found in all Hemiascomycete species, but with a very complex evolution involving repeated gene losses and duplications.

## Results and Discussion

### The relationship between the members of the *DAL5 *subfamily

**Figure 1:**
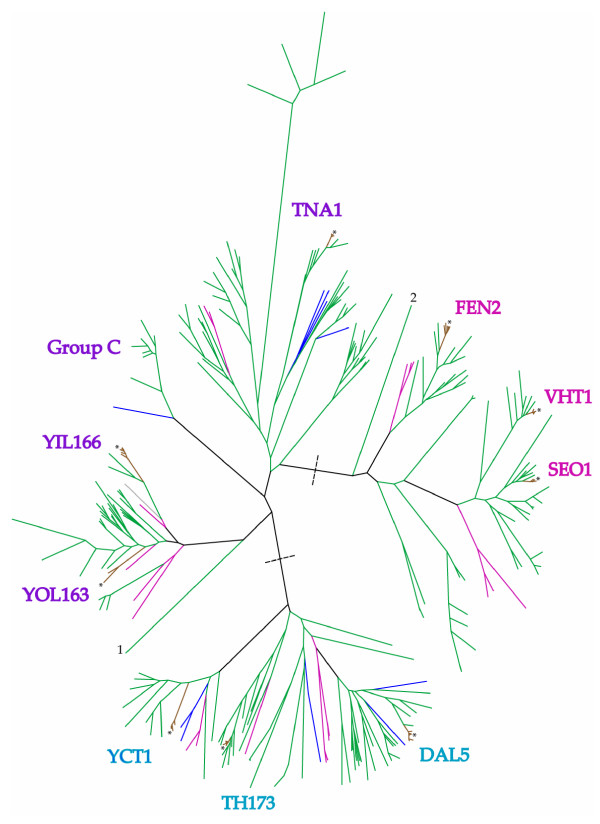
**Phylogenetic tree of all the *DAL5 *subfamily members**. The different coloures on the branches correspond to the species with the same colour in Figure 2. The gene names correspond to *S. cerevisiae *gene name and the stars (*) indicate *S. cerevisiae *branch. The dashed lines indicate the division into three major clusters, also indicated by different colours on the gene names. Nr 1 indicate the branch to *Debaryomyces hansenii *geno CAG86641.1. Nr 2 indicate the branch to *Debaryomyces hansenii *geno CAG84413.1. Group C is a well defined group with eight branches from seven different yeast species. For more detailed information see Figure 4 and the text.

The allantoate permease transporters are known to be an ancient subfamily [11], and as expected we found almost all members of the *DAL5 *group represented in the Hemiascomycete, Euascomycete and Basidiomycete taxa included in our analysis. We identified three major clusters in the phylogenetic tree of all known members of the *DAL5 *subfamily: one including the three genes *SEO1*, *VHT1 *and *FEN2 *and their orthologs, another including *YCT1*, *DAL5 *and *TH173*, and the third including YIL166c, YOL163-2w and *TNA1 *(Figure 1) for more details [see Additional file 1]. The relationships within and between these clades are consistent with those in earlier papers (e.g. [10]) with the addition of YOL163-2w, which is a pseudogene in *S. cerevisiae*, in the last group. The two genes *SEO1 *and *VHT1 *appear to be the result of an ancient duplication in an ancestor of the Saccharomycotina group. Members from the Pezizomycotina group cluster outside of the Saccharomycotina *SEO1 *and *VHT1 *clade.

**Figure 2:**
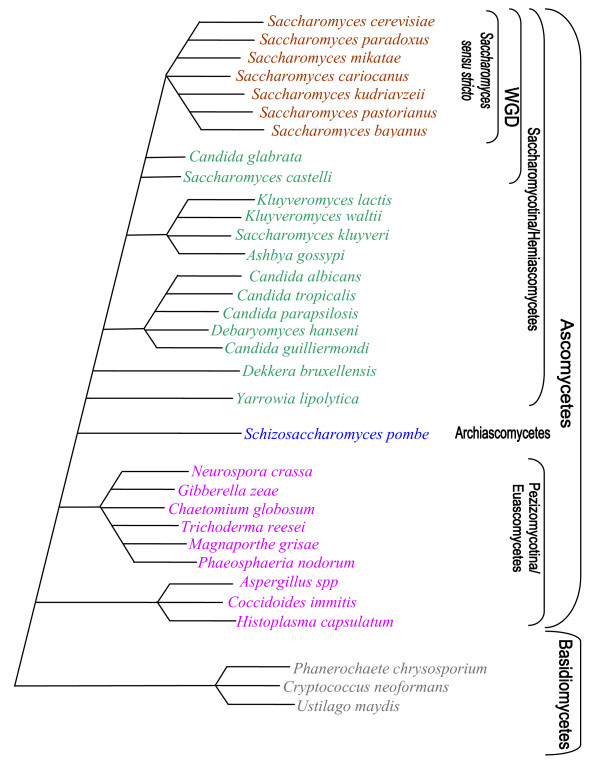
**The expected phylogeny of Ascomycetes and Basidiomycetes**. Species in the WGD cluster have undergone whole genome duplication (WGD). The colours correspond to the colours in figure 1. Adapted from [12-14].

For most genes within this subfamily, the phylogenetic relationships we observed were similar to known taxonomic relationships (compare Figure 1 and Figure 2). with Euascomycete, Basidiomycete and Hemiascomycete species each clustering together, and Basidiomycetes at the base of each tree. Within the Hemiascomycetes, we expect to find *Y. lipolytica *at the base of the clade, with *Kluyveromyces *and *Candida *species diverging subsequently, followed by the *Saccharomyces sensu stricto species *[12-14]. We observed this phylogenetic pattern overall, but with some exceptions.

### The relationship of the *DAL5 *genes

**Figure 3:**
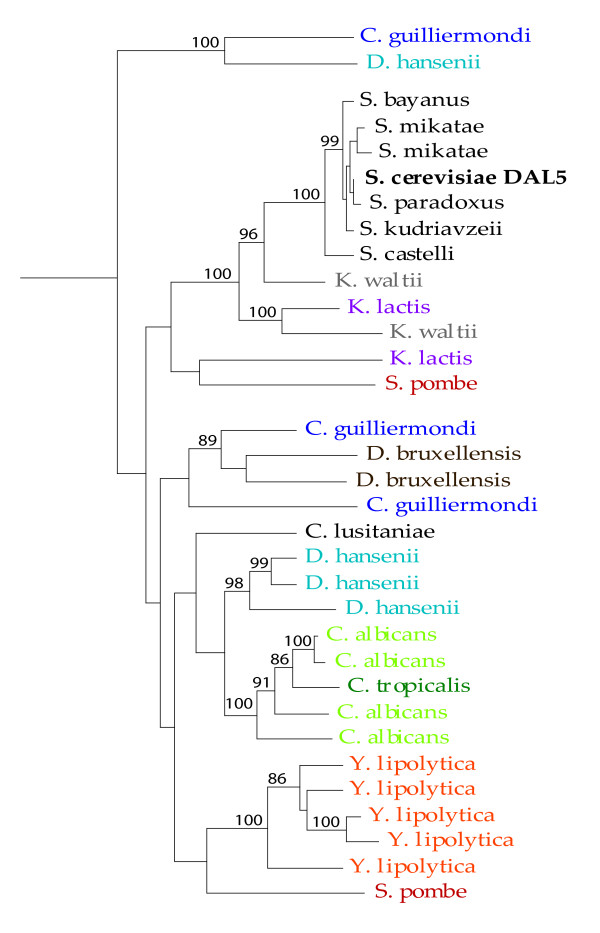
**The *DAL5 *gene tree**. Bootstrap values above 85% are indicated.

The *DAL5 *gene tree provides one exception to the expected phylogenetic relationships. *S. pombe *has two copies of this gene: one is basal to the Hemiascomycetes, as expected, but the second clusters with *K. lactis *(Figure 3). This clustering of a *S. pombe *gene with *K. lactis *has very low bootstrap support, however, and is probably artifactual. *C. guilliermondi *and *D. hansenii *both possess multiple paralogs of this gene, and each has one representative that clustered outside the Hemiascomycetes. These genes may be ancient paralogs, which may have evolved functions distinct from those described for *DAL5*.

### The relationship of the *TNA1 *genes

**Figure 4:**
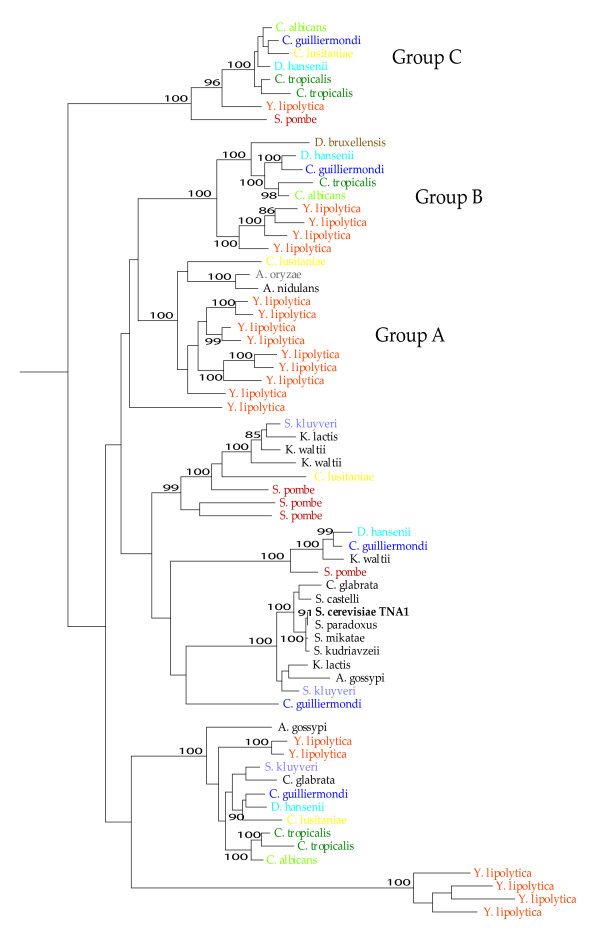
**The *TNA1 *gene tree**. Bootstrap values above 85% are indicated.

Another unexpected relationship was found in the gene tree of *TNA1*, in which four species *K. waltii*, *C. tropicalis*, *Y. lipolytica *and *S. pombe *had several gene duplicates, both recent and ancient, and spread unevenly in the gene tree. In our analyses we found five copies of this gene from *C. tropicalis *and *S. pombe *clustering at three different places in the tree and three copies from *K. waltii *in two clusters. For *Y. lipolytica *we found 20 gene copies clustering at five different places (Figure 4). A distant group of species, Group C, include only species from the pre-whole genome duplication event in the yeast clade and could constitute another gene member with a different function or specialization from *TNA1*. The paralog was obviously lost in an ancestor of the whole genome duplication lineage. This might also be the case in the groups of A and B where *Y. lipolytica *has several recent duplications.

The gene tree indicates ancient duplication of this gene, since many species have gene copies in several clusters (*C. albicans*, 3 gene copies spread over 3 clusters, *C. guilliermondi*, 5 gene copies spread over 5 clusters, *C. lusitaniae*, 4 gene copies spread over 4 clusters, *D. hansenii*, 4 gene copies spread over 4 clusters, *S. kluyveri*, 3 gene copies spread over 3 clusters, *K. lactis*, 2 gene copies spread over 2 clusters, *C. glabrata*, 2 gene copies spread over 2 clusters, *A. gossypi*, 2 gene copies spread over 2 clusters) with reciprocal losses in the other clusters which result in a non exact match of the taxonomic phylogeny.

### The relationship of the YIL166c and YOL163-2w genes

**Figure 5:**
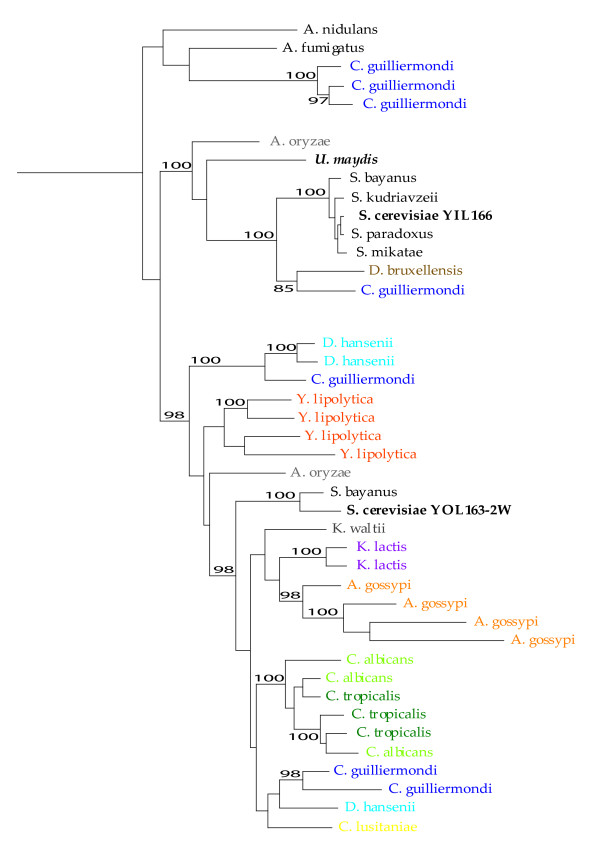
**The YIL166c and YOL163-2w gene tree**. Bootstrap values above 85% are indicated.

The evolution of the group of genes which we originally believed to be orthologs of YIL166c is more complex than we anticipated. These genes appear to be members of two paralogous clades, one consisting of YIL166c from *S. cerevisiae *and closely related genes in *the sensu stricto *species, *C. guilliermondi *and *D. bruxellensis*. Gene members from *U. maydis *and *A. oryzae *were also found in this, the smallest of the clades of the genes in the *DAL5 *subfamily (Figure 5). The second paralogous clade consists of genes from only Hemiascomycete species, including the contiguous *S. cerevisiae *genes YOL163w and YOL162w, constituting a putative single frame-shifted pseudogene. *S. cerevisiae *and *S. bayanus *were the only representatives from the *Saccharomyces sensu stricto group *that we found. More than half the members of this clade have gene duplications. Besides *Y. lipolytica*, *C. guillerimondi, C. albicans, C. tropicalis, A. gossipii *and *D. hansenii *each have two or more paralogous copies, indicating multiple recent gene duplications after species divergence. These two clades, YIL166c and YOL163-2w, have a common base represented by two Euascomycete species, *A. nidulans *and *A. fumigatus*, and three gene copies from *C. guillermondi *(Figure 5).

There are two further points of interest in this region of the tree (Figure 5). The first is the presence of a *U. maydis *(Basidiomycete) gene at the base of the Hemiascomycete YIL166c clade. Although the node has only 60% bootstrap support, the node below this is 100% supported, which results in a confident clade in which a basidiomycete is nested within ascomycetes. This relationship suggests that there has been a horizontal gene transfer event from either Euascomycetes or Hemiascomycetes to this Basidiomycete species. It will be interesting to see if a similar sequence can be detected in any other Basidiomycete species.

Secondly, while most species are represented in most gene clades, the species represented in each of these two clades (YIL166c and YOL163-2w) are substantially different. The YIL166c group contains the *sensu stricto *species, *D. bruxellensis *and *C. guilliermondi*, while the other clade contains the other species from the Hemiascomycetes. The *Saccharomyces sensu stricto *species, *C. guilliermondi *and *D. bruxellensis *have all been found in alcoholic beverages like wine [15]. The presence of these orthologous transporters in these taxa could be due to selection for retention of a transporter specially adapted to an environment of low oxygen and high ethanol concentration. It seems that the genes from these species are true orthologs with a common origin, and have either been lost from the other Hemiascomycetes or horizontally transferred into the "wine species". The gene transfer is either from a species close to *U. maydis*, which branched off before the Euascomycetes, or the gene in *U. maydis *is also a horizontally transferred gene, and the horizontal gene transfer occurred from a species in the Euascomycetes clade to *U. maydis*, and the "wine species". These hypotheses are currently speculative, and much further work would be required to investigate them further.

Another unusual behaviour of the Hemiascomycete orthologs of YIL166c is that this transporter appears to have undergone species-specific duplication in every species in our analysis. Some have been duplicated prior to speciation like *C. tropicalis *and *C. albicans*. The intraspecific duplication has resulted in two or three copies for most of the species in the trees except for *Y. lipolytica*. The pattern of gene duplication in this species is unusual: for each member of the subfamily, *Y. lipolytica *has either three or more paralogs or no copy of the gene at all. We found no orthologs of YIL166c, *YCT1*, *TH173 *and *VHT1 *in *Y. lipolytica*, but for *FEN2 *we found three copies, YOL163w four copies, *DAL5 *five copies, *SEO1 *six copies and for *TNA1 *20 copies, that met our criteria. The selective advantage of these amplifications for *Y. lipolytica *is not understood, but it could be a compensation for the loss of the other *DAL5 *transporters or a selective advantage coupled to a sudden change in the environment and/or the hydrocarbon diet. The amplification we observe of anion:cation symporters in *Y. lipolytica *has also been seen in drug:H^+^antiporter transporters and quinate:H^+^symporters [8].

### How have the gene expansions taken place?

Duplications of genes could be on the gene level, segmental, chromosomal or whole genome duplications. The duplications offer the opportunity for copies to evolve different functions, either broader or more specialized. Gene copies that provide an advantage for the organism may be preserved and go to fixation under selection. Others might lose any function, become pseudogenes and ultimately be lost from the genome. In the *DAL5 *family the number of copies various tremendously within and between species for the different gene family members. This expansion and shrinkage is most evident in *Y. lipolytica *where we found three to twenty paralogs for five of the nine subfamilies and in the others, no copy of the gene at all. The other species has also expanded into two to four copies for most genes in this subfamily. In some cases it is evident that the duplications occurred before speciation, for example see *C. albicans *and *C. tropicalis *for YOL163w, while in other cases there have been recent independent duplications in several species, see *Y. lipolytica *and *A. gossypii *in YOL163w (Figure 5). A similar pattern can be observed in the lower part of the tree *DAL5 *(Figure 3) where a few lineages (*D. hansenii, C. albicans *and *Y. lipolytica*) seem to have undergone a recent duplication and at the same time an ancestor of *S. cerevisiae *and the *sensu stricto *group deleted their copy.

Most species-specific genes are located near telomeres in *Saccharomyces sensu stricto *species [16-18]. Four (*SEO1*, *YCT1*, *DAL5 *and YIL166c) of the eight genes (nine in other species) in this subfamily are subtelomeric in *S. cerevisiae*. It is possible that the complexity that we see in many trees, especially for *Y. lipolytica *and *D. hansenii*, is a result of horizontal gene transfer coupled with intraspecific duplications. Maybe the duplications that we see are a result of specialization for different substrates or maybe even for a different function. Most of the permeases are complex in nature, with many different substrates, and for many of the species investigated here the transporter proteins are poorly studied.

## Conclusion

Yeast is one of the most important model organisms in biology research including evolution. The yeast lineage, as part of the kingdom of fungi, has revealed both horizontal transfers and whole genome duplication and is a very important group regarding new insights into evolutionary pathways [3,5,19]. Crucial genes often show an interesting evolution and transmembrane proteins are often essential for the survival of the yeast cells. The transmembrane *DAL5 *subfamily includes genes that seem to be very important due to its many duplications in almost all yeast species investigated, but it also includes genes that obviously can be deleted without any harm done to the organism. When we analysed species from the whole kingdom of fungi we found that the *DAL5 *subfamily has a very complex evolution in yeast, not seen before, with intra- and interspecific duplications and unusual relationships indicating specialization, deletions and maybe even horizontal gene transfer. We believe that the *DAL5 *subfamily will be important in future investigations of evolution in fungi, especially the evolution of transmembrane proteins and their specialization.

## Methods

### DATA

We performed two sets of BLAST searches to obtain the sequences used in this analysis. First, the protein sequences of the eight members of the *DAL5 *family from *Saccharomyces cerevisiae *(*SEO1*, *FEN2*, *VHT1*, *TNA1*, YIL166c, *DAL5*, *YCT1 *and *TH173*) were used as BLASTP queries against an NCBI genomic BLAST database of several fungal species for which whole genomes are available (*Ashbya gossypii, Candida albicans, C. glabrata, C. guilliermondii, C. lusitaniae, C. tropicalis, Debaryomyces hansenii, Kluyveromyces lactis, K. waltii, Saccharomyces bayanus, S. castellii, S. cerevisiae, S. kluyveri, S. kudriavzevii, S. mikate, S. paradoxus, Yarrowia lipolytica, Schizosaccharomyces pombe, Aspergillus fumigatus *and *A. Nidulans*). For each of these query proteins, we extracted from the BLASTP results all hits that had E-values better than 1e-6. To confirm that we had obtained all likely homologs of these genes, we compared the sequences of the hits we obtained in each query species to the database of gene family sequences made available by Jason Stajich at . Any genes in this database which were not present in our BLAST results, and were over 300 bp long, were added to our dataset.

We also used the *S. cerevisiae *genes as TBLASTN queries against a database of *Dekkera bruxellensis *contig sequences, which represent approximately 40% of the genome of *D. bruxellensis *strain CBS 2499 (Woolfit et al 2007).

### Phylogenetic analysis

We initially treated each of the query genes, together with their sets of hits, as separate datasets. As preliminary analyses suggested that the genes *SEO1 *and *VHT1 *represented a Hemiascomycete-specific duplication, the results for these two genes were combined, leaving us with seven datasets.

For each of the datasets, the protein sequences were aligned using T_Coffee. The alignments were edited by hand, and any regions of the sequences for which homology could not be confidently established were removed producing alignments of between 410 and 526 residues in length (*SEO1*-*VHT1*: 495aa, *FEN2*: 449aa, *DAL5*: 416aa, *YCT1*: 488aa, *TH173*: 486aa, *TNA1*: 410aa, YIL166C:526aa). Phylogenetic trees were constructed for each dataset using PHYML, using the JTT model of substitution, and a gamma distribution of rates with four categories. The gamma parameter was estimated from the data. One thousand bootstrap replicates of each tree were run, using the same model parameters.

We then combined all sequences into a single alignment. The seven previously aligned datasets were used as "profiles" and aligned against one another using the profile comparison function in T_Coffee. The resulting alignment of 650 residues was checked manually, and phylogenetic analyses were performed as described above. To confirm that the step-wise method of alignment had not affected our results, gaps were removed, all sequences were realigned using Muscle, and the phylogenetic analysis was repeated. These results were not qualitatively different.

## Authors' contributions

LIH did the initial phylogenetic studies, tree-analyses, manuscript and figure preparation. MEW did the data handling and extensive phylogenetic analyses. MAH wrote a computer program for fast accessing of homologous sequences from the net. JUP was involved in manuscript editing and tree-analyses. All authors read and approved the manuscript.

## Supplementary Material

Additional file 1 "DAL5 family gene tree", the figure displays a phylogenetic tree which contains all sequences used with their systematic names and is the original tree from which Figure 1 is done. Click here for file

## References

[B1] Paulsen IT, Sliwinski MK, Nelissen B, Goffeau A, Saier Jr MH (1998). Unified inventory of established and putative transporters encoded within the complete genome of Saccharomyces cerevisiae. FEBS Letters.

[B2] De Hertogh B, Carvajal E, Talla E, Dujon B, Baret P, Goffeau A (2002). Phylogenetic classification of transporters and other membrane proteins from Saccharomyces cerevisiae. Funct Integr Genomics.

[B3] Dujon B, Sherman D, Fischer G, Durrens P, Casaregola S, Lafontaine I, De Montigny J, Marck C, Neuveglise C, Talla E, Goffard N, Frangeul L, Aigle M, Anthouard V, Babour A, Barbe V, Barnay S, Blanchin S, Beckerich JM, Beyne E, Bleykasten C, Boisrame A, Boyer J, Cattolico L, Confanioleri F, De Daruvar A, Despons L, Fabre E, Fairhead C, Ferry-Dumazet H, Groppi A, Hantraye F, Hennequin C, Jauniaux N, Joyet P, Kachouri R, Kerrest A, Koszul R, Lemaire M, Lesur I, Ma L, Muller H, Nicaud JM, Nikolski M, Oztas S, Ozier-Kalogeropoulos O, Pellenz S, Potier S, Richard GF, Straub ML, Suleau A, Swennen D, Tekaia F, Wesolowski-Louvel M, Westhof E, Wirth B, Zeniou-Meyer M, Zivanovic I, Bolotin-Fukuhara M, Thierry A, Bouchier C, Caudron B, Scarpelli C, Gaillardin C, Weissenbach J, Wincker P, Souciet JL (2004). Genome evolution in yeasts. Nature.

[B4] Scannell DR, Byrne KP, Gordon JL, Wong S, Wolfe KH (2006). Multiple rounds of speciation associated with reciprocal gene loss in polyploid yeasts. Nature.

[B5] Dujon B (2006). Yeasts illustrate the molecular mechanisms of eukaryotic genome evolution. Trends Genet.

[B6] Maclean RC (2005). Ecological genetics: the decline and fall of a metabolic pathway in yeast. Heredity.

[B7] Hittinger CT, Rokas A, Carroll SB (2004). Parallel inactivation of multiple GAL pathway genes and ecological diversification in yeasts. Proc Natl Acad Sci U S A.

[B8] De Hertogh B, Hancy F, Goffeau A, Baret PV (2006). Emergence of Species-Specific Transporters During Evolution of the Hemiascomycete Phylum. Genetics.

[B9] Diffels JF, Seret ML, Goffeau A, Baret PV (2006). Heavy metal transporters in Hemiascomycete yeasts. Biochimie.

[B10] Nelissen B, De Wachter R, Goffeau A (1997). Classification of all putative permeases and other membrane plurispanners of the major facilitator superfamily encoded by the complete genome of Saccharomyces cerevisiae. FEMS Microbiol Rev.

[B11] Heckman DS, Geiser DM, Eidell BR, Stauffer RL, Kardos NL, Hedges SB (2001). Molecular Evidence for the Early Colonization of Land by Fungi and Plants. Science.

[B12] Fitzpatrick D, Logue M, Stajich J, Butler G (2006). A fungal phylogeny based on 42 complete genomes derived from supertree and combined gene analysis. BMC Evolutionary Biology.

[B13] Wolfe KH (2006). Comparative genomics and genome evolution in yeasts. Philos Trans R Soc Lond B Biol Sci.

[B14] Kuramae EE, Robert V, Snel B, Boekhout T (2006). Conflicting phylogenetic position of Schizosaccharomyces pombe. Genomics.

[B15] Martorell P, Barata A, Malfeito-Ferreira M, Fernandez-Espinar MT, Loureiro V, Querol A (2006). Molecular typing of the yeast species Dekkera bruxellensis and Pichia guilliermondii recovered from wine related sources. Int J Food Microbiol.

[B16] Hall C, Brachat S, Dietrich FS (2005). Contribution of horizontal gene transfer to the evolution of Saccharomyces cerevisiae. Eukaryot Cell.

[B17] Kellis M, Patterson N, Endrizzi M, Birren B, Lander ES (2003). Sequencing and comparison of yeast species to identify genes and regulatory elements. Nature.

[B18] Cliften P, Sudarsanam P, Desikan A, Fulton L, Fulton B, Majors J, Waterston R, Cohen BA, Johnston M (2003). Finding functional features in Saccharomyces genomes by phylogenetic footprinting. Science.

[B19] Cliften PF, Fulton RS, Wilson RK, Johnston M (2006). After the Duplication: Gene Loss and Adaptation in Saccharomyces Genomes. Genetics.

[B20] Rai R, Genbauffe F, Lea HZ, Cooper TG (1987). Transcriptional regulation of the DAL5 gene in Saccharomyces cerevisiae. J Bacteriol.

[B21] Rai R, Genbauffe FS, Cooper TG (1988). Structure and transcription of the allantoate permease gene (DAL5) from Saccharomyces cerevisiae. J Bacteriol.

[B22] Stolz J, Hoja U, Meier S, Sauer N, Schweizer E (1999). Identification of the plasma membrane H+-biotin symporter of Saccharomyces cerevisiae by rescue of a fatty acid-auxotrophic mutant. J Biol Chem.

[B23] Marcireau C, Joets J, Pousset D, Guilloton M, Karst F (1996). FEN2: a gene implicated in the catabolite repression-mediated regulation of ergosterol biosynthesis in yeast. Yeast.

[B24] Shakoury-Elizeh M, Tiedeman J, Rashford J, Ferea T, Demeter J, Garcia E, Rolfes R, Brown PO, Botstein D, Philpott CC (2004). Transcriptional remodeling in response to iron deprivation in Saccharomyces cerevisiae. Mol Biol Cell.

[B25] Klebl F, Zillig M, Sauer N (2000). Transcription of the yeast TNA1 gene is not only regulated by nicotinate but also by p-aminobenzoate. FEBS Lett.

[B26] Llorente B, Dujon B (2000). Transcriptional regulation of the Saccharomyces cerevisiae DAL5 gene family and identification of the high affinity nicotinic acid permease TNA1 (YGR260w). FEBS Lett.

[B27] Boer VM, de Winde JH, Pronk JT, Piper MD (2003). The genome-wide transcriptional responses of Saccharomyces cerevisiae grown on glucose in aerobic chemostat cultures limited for carbon, nitrogen, phosphorus, or sulfur. J Biol Chem.

[B28] Giaever G, Chu AM, Ni L, Connelly C, Riles L, Veronneau S, Dow S, Lucau-Danila A, Anderson K, Andre B, Arkin AP, Astromoff A, El-Bakkoury M, Bangham R, Benito R, Brachat S, Campanaro S, Curtiss M, Davis K, Deutschbauer A, Entian KD, Flaherty P, Foury F, Garfinkel DJ, Gerstein M, Gotte D, Guldener U, Hegemann JH, Hempel S, Herman Z, Jaramillo DF, Kelly DE, Kelly SL, Kotter P, LaBonte D, Lamb DC, Lan N, Liang H, Liao H, Liu L, Luo C, Lussier M, Mao R, Menard P, Ooi SL, Revuelta JL, Roberts CJ, Rose M, Ross-Macdonald P, Scherens B, Schimmack G, Shafer B, Shoemaker DD, Sookhai-Mahadeo S, Storms RK, Strathern JN, Valle G, Voet M, Volckaert G, Wang CY, Ward TR, Wilhelmy J, Winzeler EA, Yang Y, Yen G, Youngman E, Yu K, Bussey H, Boeke JD, Snyder M, Philippsen P, Davis RW, Johnston M (2002). Functional profiling of the Saccharomyces cerevisiae genome. Nature.

[B29] Nelissen B, Mordant P, Jonniaux JL, De Wachter R, Goffeau A (1995). Phylogenetic classification of the major superfamily of membrane transport facilitators, as deduced from yeast genome sequencing. FEBS Lett.

[B30] Mojzita D, Hohmann S (2006). Pdc2 coordinates expression of the THI regulon in the yeast Saccharomyces cerevisiae. Mol Genet Genomics.

[B31] Isnard AD, Thomas D, Surdin-Kerjan Y (1996). The study of methionine uptake in Saccharomyces cerevisiae reveals a new family of amino acid permeases. J Mol Biol.

[B32] Ahmed Khan S, Zhang N, Ismail T, El-Moghazy AN, Butt A, Wu J, Merlotti C, Hayes A, Gardner DC, Oliver SG (2000). Functional analysis of eight open reading frames on chromosomes XII and XIV of Saccharomyces cerevisiae. Yeast.

