# Supplementary figures and images for: Complex evolution of the DAL5 transporter family
Source: BMC Genomics. 2008 Apr 11;9:164. doi: 10.1186/1471-2164-9-164 (PMC2329640; doi:10.1186/1471-2164-9-164)

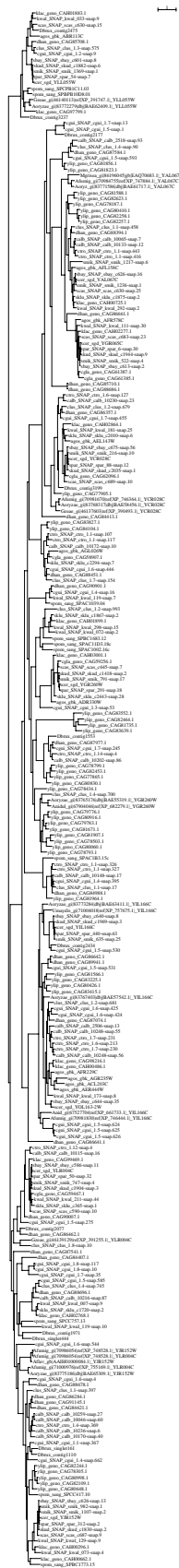

Supplement: Additional file 1 — "DAL5 family gene tree", the figure displays a phylogenetic tree which contains all sequences used with their systematic names and is the original tree from which Figure 1 is done. [file 1471-2164-9-164-S1.pdf]
